# Supplementary material for: Water conservation benefits of urban heat mitigation
Source: Nat Commun. 2017 Oct 20;8:1072. doi: 10.1038/s41467-017-01346-1 (PMC5651875; doi:10.1038/s41467-017-01346-1)
Supplement: Supplementary file 1 — Supplementary Information [file 41467_2017_1346_MOESM1_ESM.pdf]

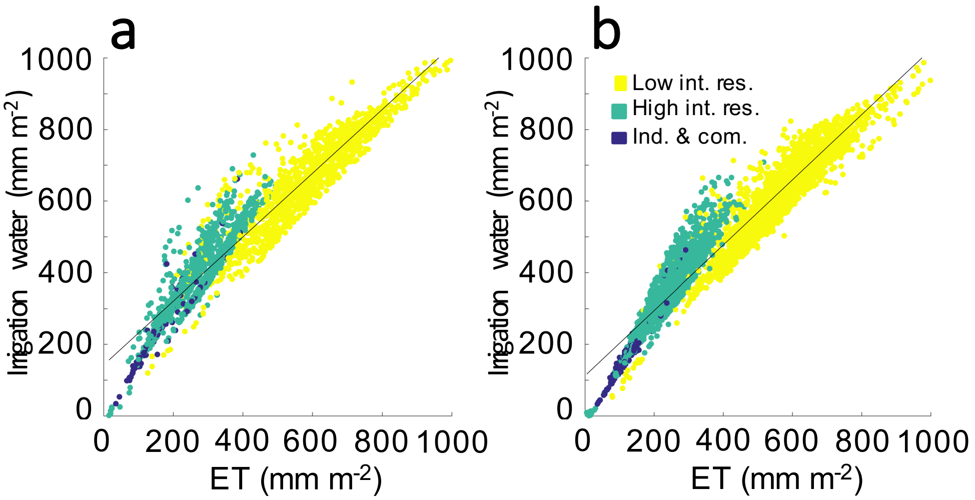

Supplementary Figure 1. Correlation between simulated irrigation water and evapotranspiration (ET) for NorCal (**a**) and SoCal (**b**) for Control simulations. Each value represents the acumulated irrigation water consumption and ET over June-October averaged over 2001-2015 for each urban grid-cell. The circle colors in illustrate the urban type: low intensity residential (low int. res.), high intensity residential (high int. res.), and industrial and commercial (ind. & com.). The black lines illustrate best linear fit (in a least-squares sense) to the data.

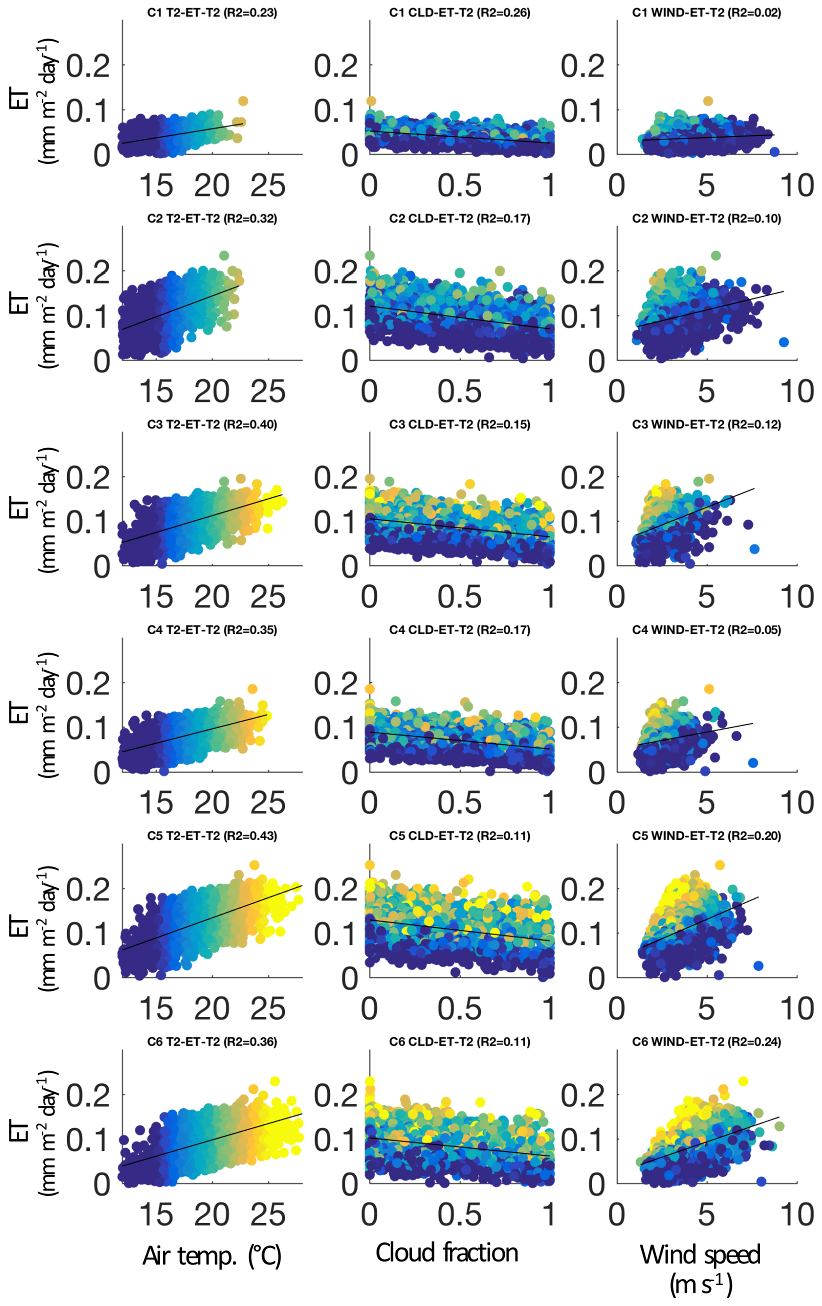

Supplementary Figure 2. Correspondence between simulated daily evapotranspiration (ET) versus 2-m air temperature (left column), cloud fraction (middle column), and wind speed (right column) for San Francisco (C1), San Mateo (C2), Santa Clara (C3), Alameda (C4), Contra Costa (C5), and Solano (C6), from top to bottom, respectively. Circles represent daily means over urban surfaces for June-October of 2001-2015. The circle colors illustrate the daily air temperature. The balck lines show the best linear fit (in a least-squares sense) to the data.

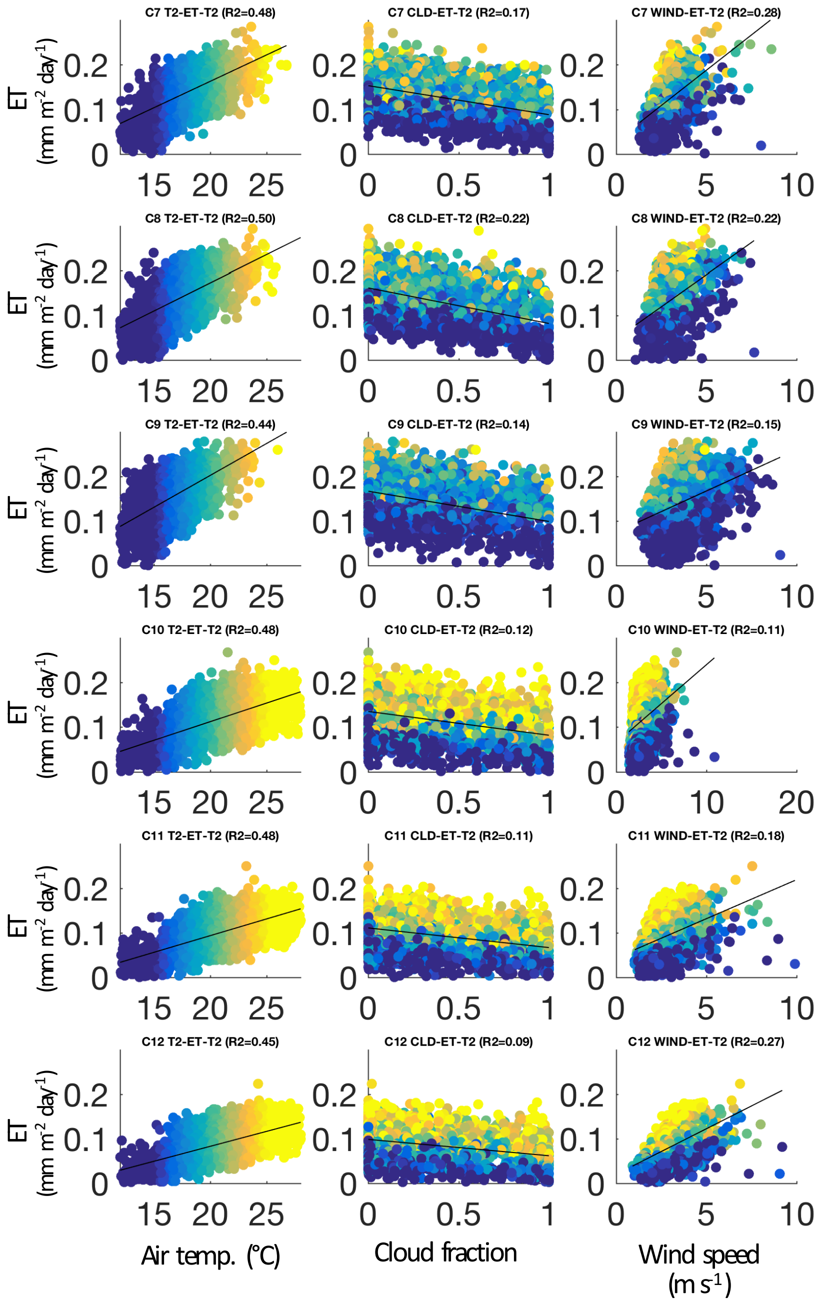

Supplementary Figure 3. Correspondence between simulated daily evapotranspiration (ET) versus 2-m air temperature (left column), cloud fraction (middle column), and wind speed (right column) for Napa (C7), Sonoma (C8), Marin (C9), Placer (C10), Sacramento (C11), and San Joaquin (C12), from top to bottom, respectively. Circles represent daily means over urban surfaces for June-October of 2001-2015. The circle colors illustrate the daily air temperature. The balck lines show the best linear fit (in a least-squares sense) to the data.

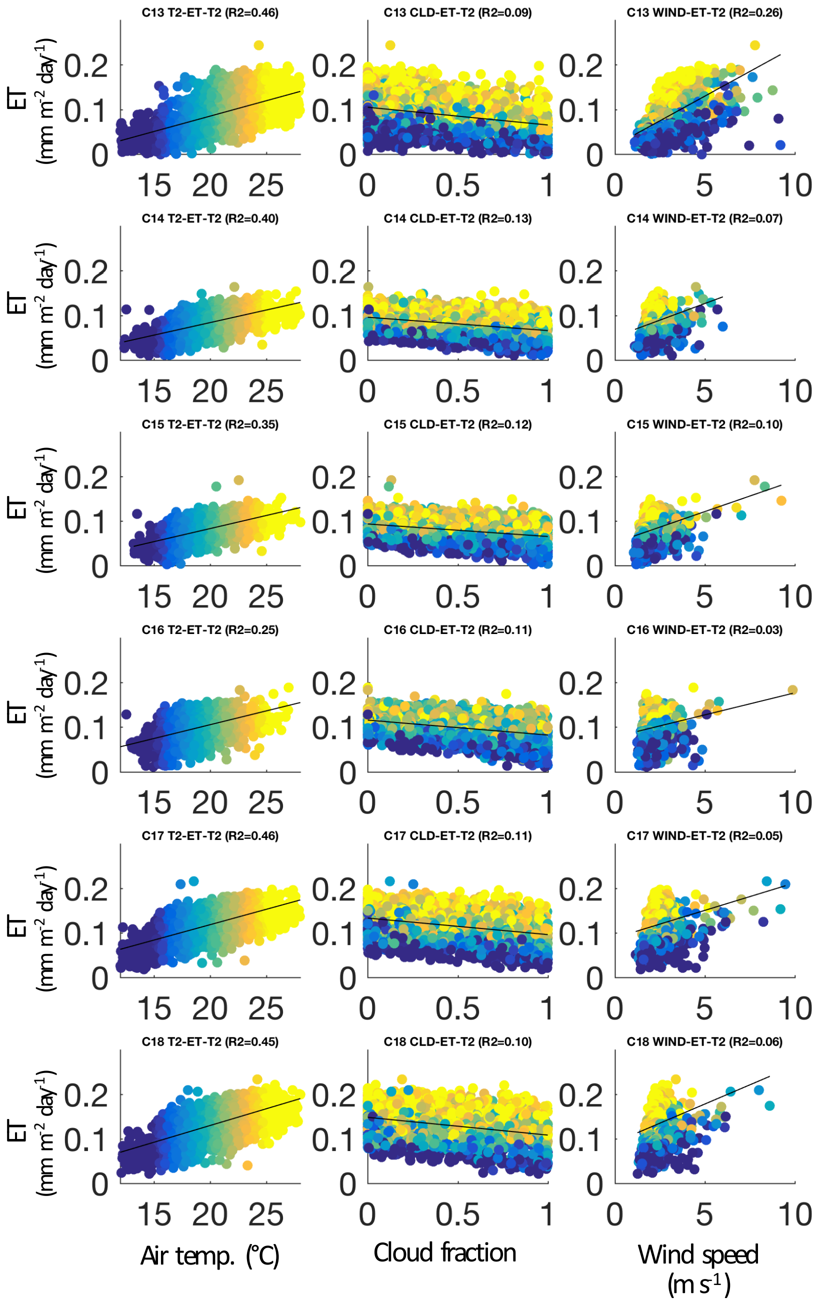

Supplementary Figure 4. Correspondence between simulated daily evapotranspiration (ET) versus 2-m air temperature (left column), cloud fraction (middle column), and wind speed (right column) for Stanislaus (C13), Los Angeles (C14), Orange (C15), San Diego (C16), San Bernardino (C17), and Riverside (C18), from top to bottom, respectively. Circles represent daily means over urban surfaces for June-October of 2001-2015. The circle colors illustrate the daily air temperature. The balck lines show the best linear fit (in a least-squares sense) to the data.

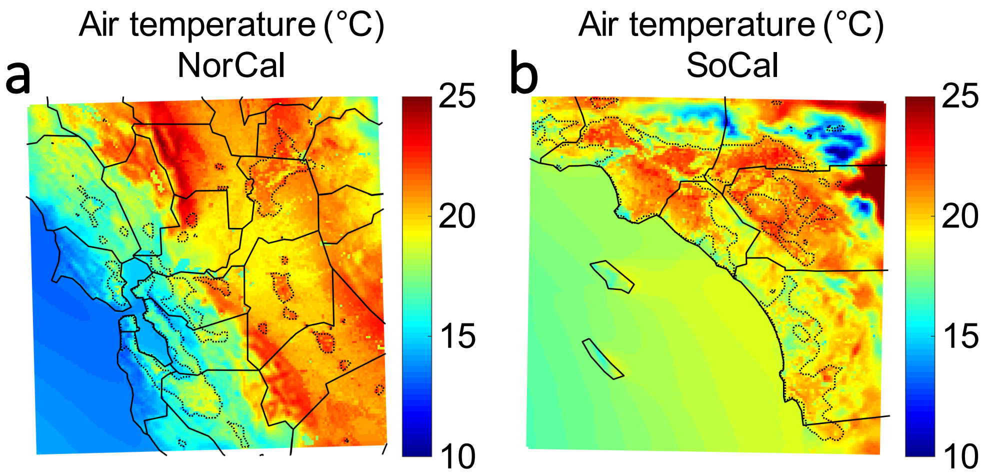

Supplementary Figure 5. Simulated 2-m air temperature from Control simulations for NorCal (a) and SoCal (b). Values represent averages over June-October of 2001-2015. The solid black lines illustrate the boundaries of the 18 urban counties that are captured in the model domains.

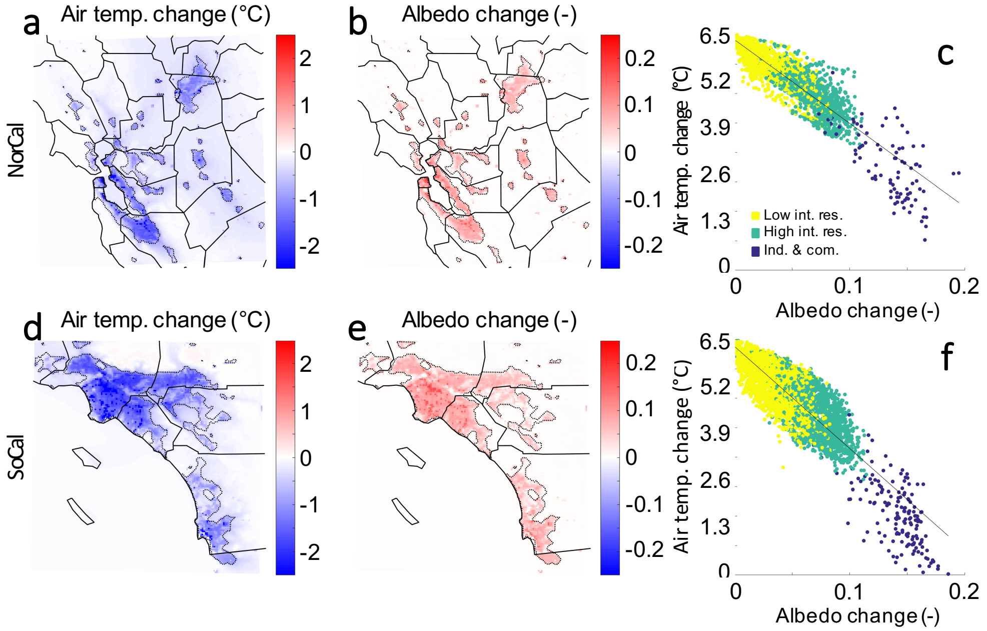

Supplementary Figure 6. Simulated cool-roof-indcued 2-m air temperature change (a and d), albedo change (b and e) and corelation between them (c and f). Values represent averages over June-October of 2001-2015. The solid black lines in (a-b and d-e) illustrate the boundaries of the 18 urban counties that are captured in the model domains. The boundaries of urban surfaces are illustrated by dotted black lines in (a-b and d-e). Note that only changes that are statistically distinguishable from zero at 95% confidence interval are shown. The circle colors in (c and f) illustrate the urban types of low intensity residential (low int. res.), high intensity residential (high int. res.), and industrial and commercial (ind. & com.). The balck lines in (c and f) show the best linear fit (in a least-squares sense) to the data.

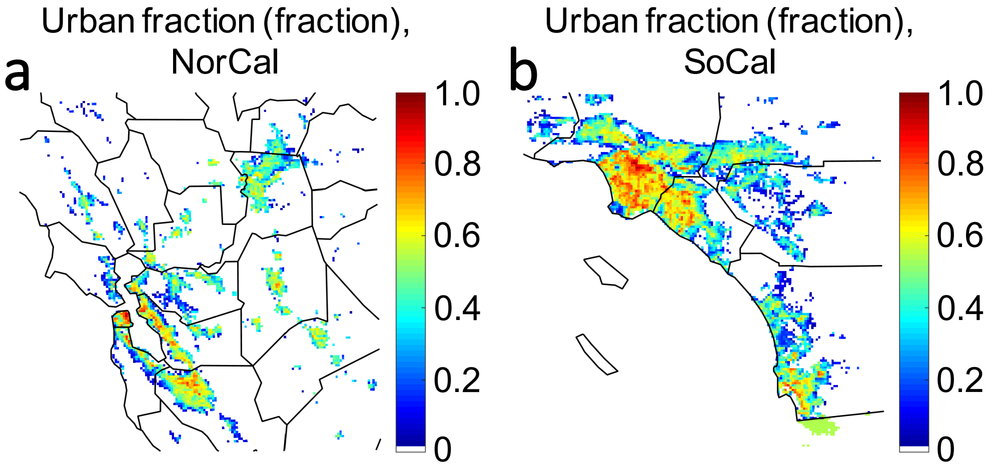

Supplementary Figure 7. Urban fraction for NorCal (a) and SoCal (b). The black lines illustrate the boundaries of the 18 urban counties that are captured in the model domains.

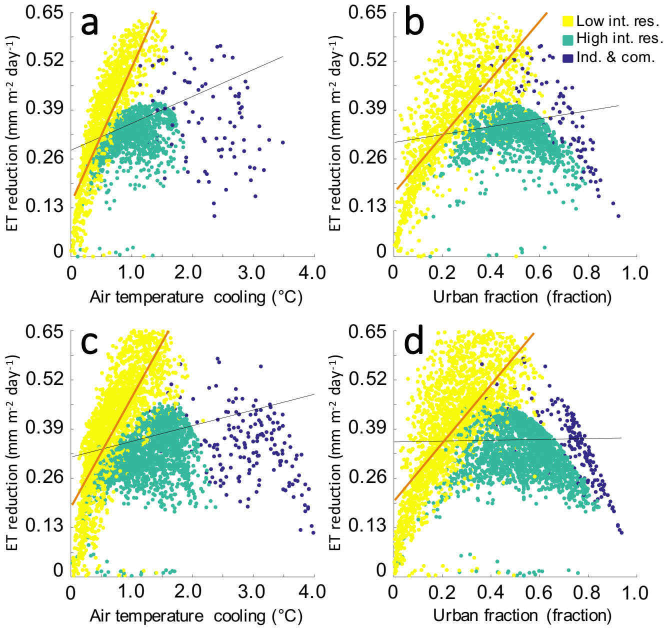

Supplementary Figure 8. Correspondence between simulated cool-roof-induced evapotranspiration (ET) reduction versus air tempetaure cooling and urban fraction for NorCal (a-b) and SoCal (c-d). Each circle represents a mean value over June-October of 2001-2015 for one urban grid-cell. The circle colors illustrate the urban types of low intensity residential (low int. res.), high intensity residential (high int. res.), and industrial and commercial (ind. & com.). The balck lines show the best linear fit (in a least-squares sense) across all urban grid-cells. The orange lines show the best linear fit to low intensity residential grid-cells only.

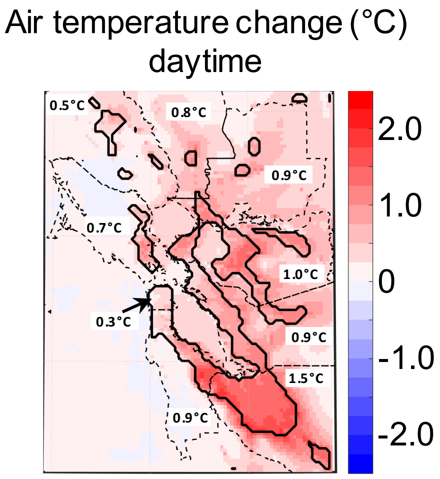

Supplementary Figure 9. Simulated 2-m air temperature changes at 2pm induced by a complete cessation of irrigation for the months of June-October of 2012, 2013, and 2014. The black dashed and solid lines illustrate the boundaries of the 9 counties and urban surfaces in the San Francisco Bay Area, respectively. Mean air temperature changes for urban surfaces in the 9 counties are indicated. Note that only changes that are statistically distinguishable from zero at 95% confidence interval are shown.

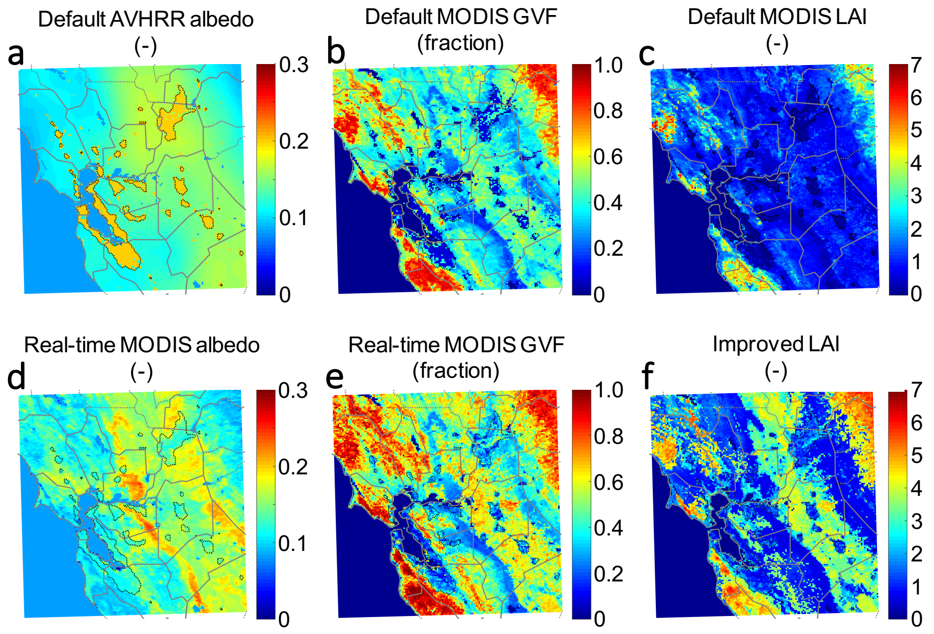

Supplementary Figure 10. Albedo, green vegetation fraction (GVF), and leaf area index (LAI) maps based on climatalogical data used in defaut WRF-UCM (a-c) and real-time MODIS data (d-f) for NorCal. Note that LAI values are not directly aquired from MODIS data. But they are tabulated values that rely on MODIS-based GVF for inter-annual and monthly variabilities. Values represent averages over June-October of 2001-2015. The grey lines illustrate the boundaries of the urban counties that are captured in the model domain.

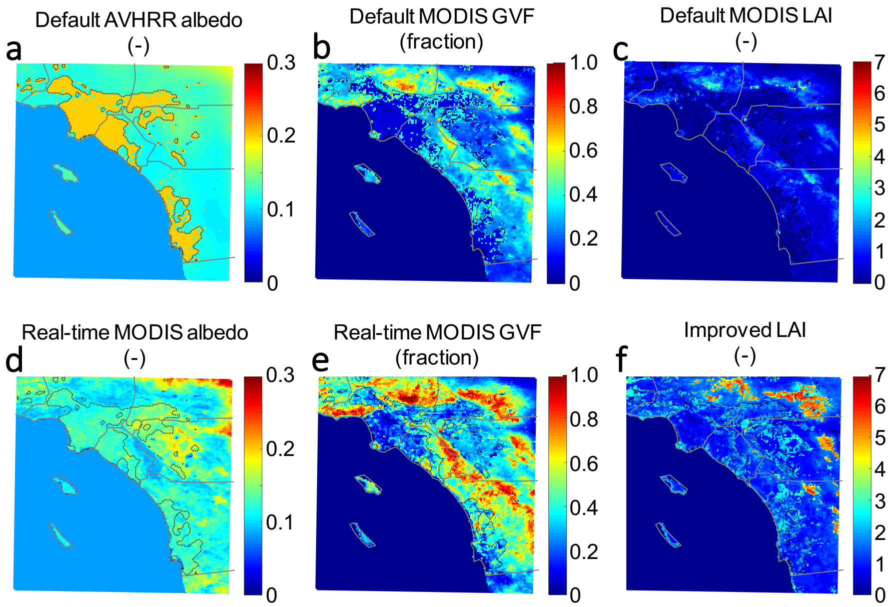

Supplementary Figure 11. Albedo, green vegetation fraction (GVF), and leaf area index (LAI) maps based on climatalogical data used in defaut WRF-UCM (a-c) and real-time MODIS data (d-f) for SoCal. Note that LAI values are not directly aquired from MODIS data. But they are tabulated values that rely on MODIS-based GVF for inter-annual and monthly variabilities. Values represent averages over June-October of 2001-2015. The grey lines illustrate the boundaries of the urban counties that are captured in the model domain.

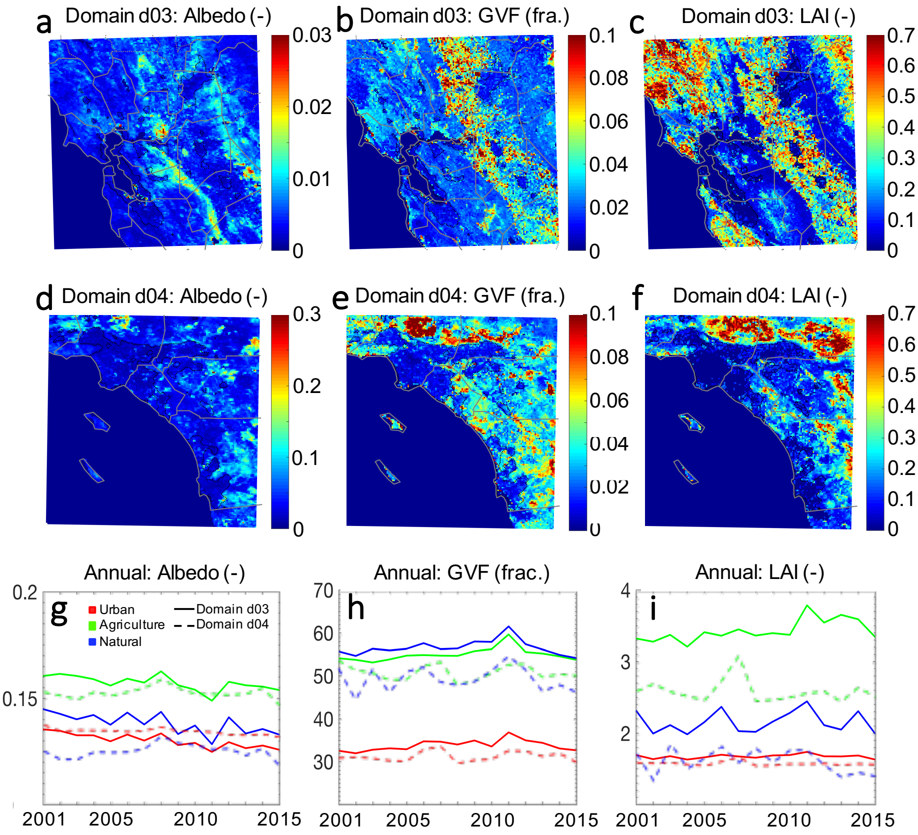

Supplementary Figure 12. Absolute mean deviation maps of albedo, green vegetation fraction (GVF), and leaf area index (LAI) based on 15 years (2001-2015) of MODIS-based data used in the improved WRF-UCM for NorCal (a-c) and SoCal (d-f). The annual averages over urban (red), agricultrual (green), and natural (blue) areas are also presented (g-i) for NorCal (solid) and SoCal (dashed). Values are calculated over June-October for each year. The grey lines in top two rows illustrate the boundaries of the 18 urban counties that are captured in the model domains.

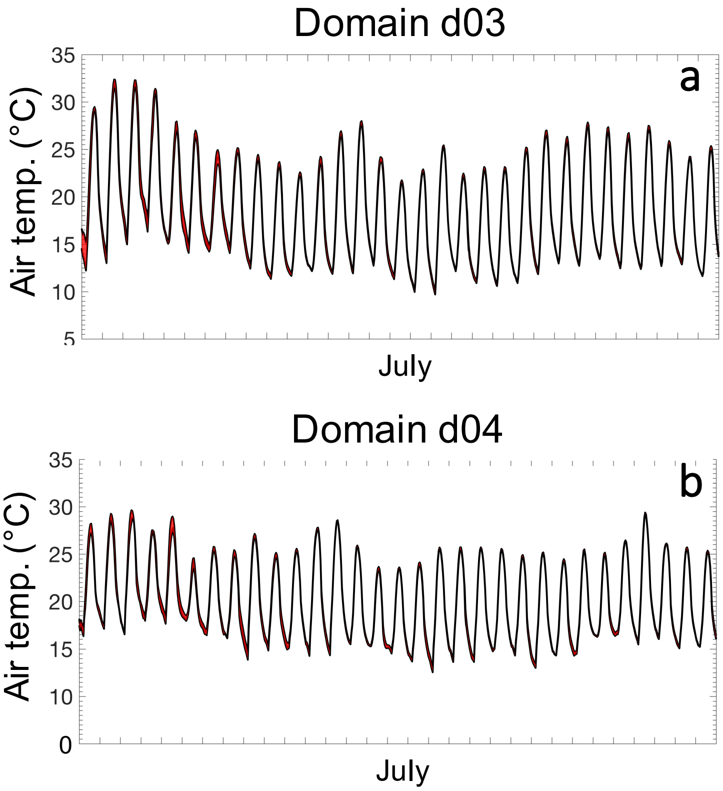

Supplementary Figure 13. Simulated hourly 2-m air temperature over July of 2001 for NorCal (a) and SoCal (b). The simulated averages over urban areas from 3 ensemble members with different initial start times are presented by black lines. Red fillings highlight the defference between simulated temperatures from these ensemble members.

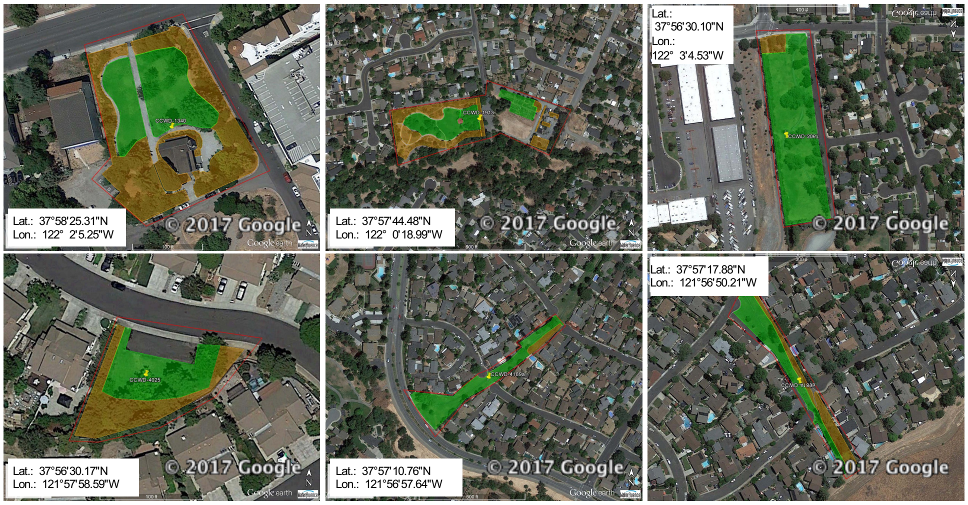

Supplementary Figure 14. Google Earth areal images and locations of the 6 parks (irrigation only consumers) used for validation of irrigation scheme implemented in WRF-UCM. The irrigated surfaces are colored (green for turf grass and orange for trees). The latitude (Lat.) and longtitude (Lon.) of parks are also indicated. Map data: Google. The areas of the parks are measured using Google Earth Pro.

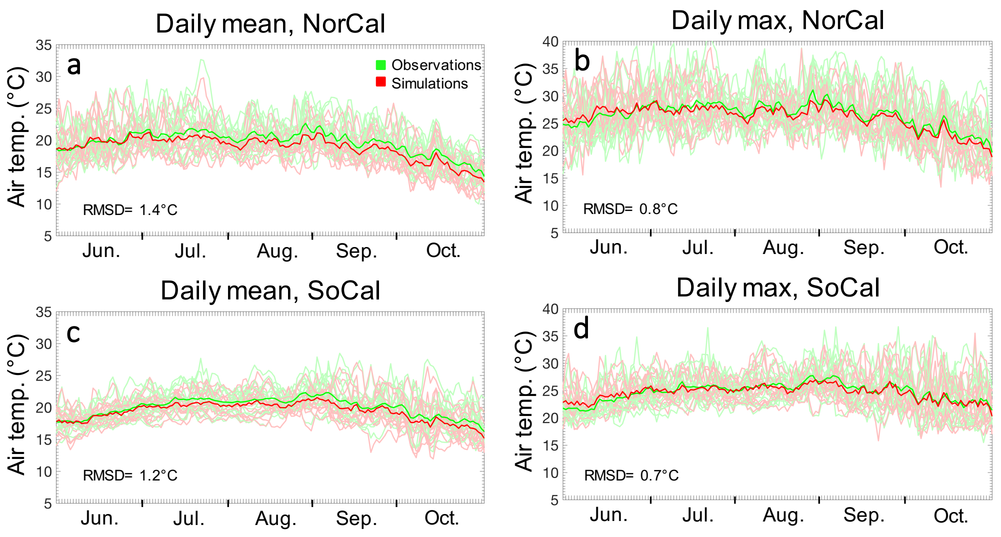

Supplementary Figure 15. Simulated (red) and observed (green) daily mean (a and c) and max (b and d) 2-m air temperature over June-October averaged over 2001-2015 for NorCal (a-b) and SoCal (c-d). The observed and simulated values represent averages of the measurements from 35 NCDC stations shown in Figure 1 and the WRF-UCM predictions over the corresponding grid-cells, respectively. The faided lines represent the values from each year from 2001-2015.

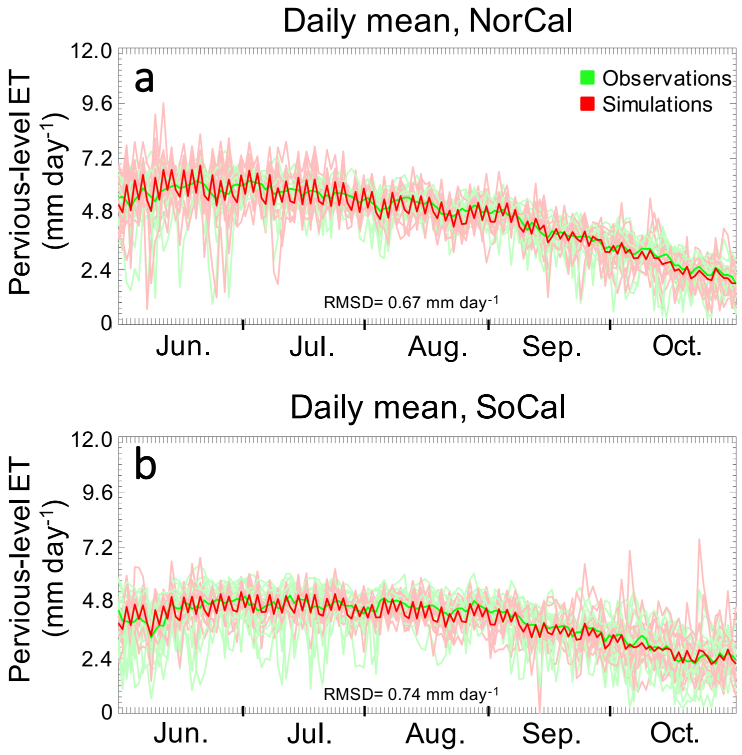

Supplementary Figure 16. Simulated (red) and observed (green) daily pervious-level evapotranspiration (ET) over June-October averaged over 2001-2015 for NorCal (a) and Socal (b). The observed and simulated values represent averages of the measurements from 34 CIMIS stations shown in the Figure 1 and the WRF-UCM predictions over the corresponding grid-cells, respectively. The faided lines represent the values from each year from 2001-2015.

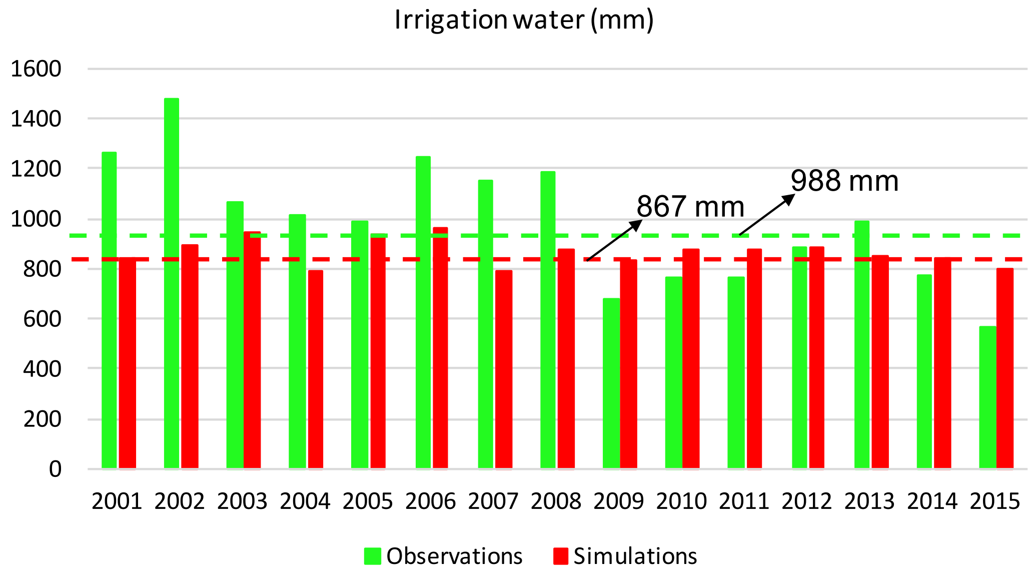

Supplementary Figure 17. Simulated (red) and observed (green) total irrigation water use over June-October for 2001-2015. The observed and simulated values represent averages of the measurements from 6 parks (irrigation only consumers) in the Contra Costa county shown in the supplementary Figure 14 and the WRF-UCM predictions over the corresponding grid-cells, respectively. The dashed lines represent the averages over 15 years of data from WRF-UCM simulations and observations.
